# Acoustically Driven Cell-Based Microrobots for Targeted Tumor Therapy

**DOI:** 10.3390/pharmaceutics14102143

**Published:** 2022-10-09

**Authors:** Hiep Xuan Cao, Van Du Nguyen, Daewon Jung, Eunpyo Choi, Chang-Sei Kim, Jong-Oh Park, Byungjeon Kang

**Affiliations:** 1 School of Mechanical Engineering, Chonnam National University, Gwangju 61186, Korea; 2 Korea Institute of Medical Microrobotics, Gwangju 61011, Korea; 3 College of AI Convergence, Chonnam National University, Gwangju 61186, Korea; 4 Graduate School of Data Science, Chonnam National University, Gwangju 61186, Korea

**Keywords:** acoustic manipulation, cell-based microrobot, targeted drug delivery

## Abstract

Targeted drug delivery using microrobots manipulated by an external actuator has significant potential to be a practical approach for wireless delivery of therapeutic agents to the targeted tumor. This work aimed to develop a novel acoustic manipulation system and macrophage-based microrobots (Macbots) for a study in targeted tumor therapy. The Macbots containing superparamagnetic iron oxide nanoparticles (SPIONs) can serve as drug carriers. Under an acoustic field, a microrobot cluster of the Macbots is manipulated by following a predefined trajectory and can reach the target with a different contact angle. As a fundamental validation, we investigated an in vitro experiment for targeted tumor therapy. The microrobot cluster could be manipulated to any point in the 4 × 4 × 4 mm region of interest with a position error of less than 300 μm. Furthermore, the microrobot could rotate in the O-XY plane with an angle step of 45 degrees without limitation of total angle. Finally, we verified that the Macbots could penetrate a 3D tumor spheroid that mimics an in vivo solid tumor. The outcome of this study suggests that the Macbots manipulated by acoustic actuators have potential applications for targeted tumor therapy.

## 1. Introduction

In the last few decades, there has been a rapid development of three-dimensional (3D) printing and nanotechnology that enable the fabrication of on-demand artificial machines at a micro/nanoscale. As widely investigated, micro/nano machines have a remarkable potential to perform various tasks in several media [1,2,3,4,5,6,7,8]. These micro/nano machines are capable of wireless manipulation controlled by external power sources [9,10,11], self-propulsion [12,13,14,15], and hybrid propulsion [16,17,18]. These external power sources use different mechanisms including optical, magnetic, hydrodynamic, electric, and acoustic fields. Each method has its advantages and disadvantages in manipulating micro/nanomachines. Optical manipulation provides very precise control with the highest degree of spatial resolution, but the penetration depth is limited. Moreover, a high-powered laser system is generally used, which can damage the living object [19,20,21]. The magnetic manipulation method has the advantages of long penetration depth, a large region of interest (ROI), and powerful torque. However, this method requires magnetic characteristics, a high current power source, and a large space to set up the system [22,23,24]. Moreover, the magnetic field is globally affected by the whole ROI, which can cause difficulty for selective control among multiple objects [25,26,27]. The hydrodynamic manipulation method may be the simplest method to manipulate micro/nano machines by using fluid flow control within a microchannel. However, this approach requires a specific microchannel platform with a flow control method for a specific object, and the controllability of nanoparticles is limited [28,29,30]. The electric method uses electrophoretic and dielectrophoretic forces to trap and manipulate particles with the widest range from 1 μm to 1 mm compared with other approaches [31,32,33]. However, it requires a low-conductivity environment, cell polarizability, and specific particle materials. Among these approaches, acoustic manipulation has many advantages in the wireless manipulation of micro/nano machines for medical applications. First, acoustic energy can be transmitted through the human body with long penetration depths, which can reach all organs in the body. Second, the acoustic manipulation method can trap a nanometer- to millimeter-sized object at a specific point; thus, it can gather or separate objects without requiring the specific characteristic of object materials. Third, acoustic energy usage has a long history, and it is a safe technology for the human body as well as a biocompatible energy source [7,34,35,36]. Table 1 shows the summary of previously reported external powered platforms for wireless particle manipulation with certain operating parameters and system requirements.

Acoustic actuation generally consists of one to multiple piezoelectric transducers that generate a pressure field to trap and manipulate the object in low acoustic impedance media, commonly known as acoustic tweezers. Considering the ability to precisely control a single agent [37,38,39] as well as a cluster of agents [31,36,40] at different length scales, safety, and biocompatibility, acoustically-powered systems are becoming versatile and efficient platforms in diverse applications. Many approaches have been reported for levitating and manipulating an object in air [34,41], microfluidic channels [42,43,44], and in vitro experiments [40,45,46,47,48]. The potential of using acoustic actuators in medical applications has been widely investigated. Most clinical applications require an external power system that is compatible with biological media and suitable to operate on the human body. In addition, various medical applications require the use of micro/nano agents as drug containers.

Owing to the ineffectiveness of drug delivery systems employing drug-loaded nanoparticles with enhanced permeability and retention effects [49], many researchers have focused on the development of cell-based drug delivery systems, that is, actively delivering drugs to the targeted sites using cells [50,51,52]. Macrophages are immune cells that are a part of the mononuclear phagocyte system. Compared with other cell types used in drug delivery systems, such as bacteria and stem cells, macrophages have a better ability to engulf nanoparticles using a process called phagocytosis. In addition, they possess the ability to target tumors, known as tumor-associated macrophages, and form approximately 70% of a tumor mass [53,54].

**Figure 1:**
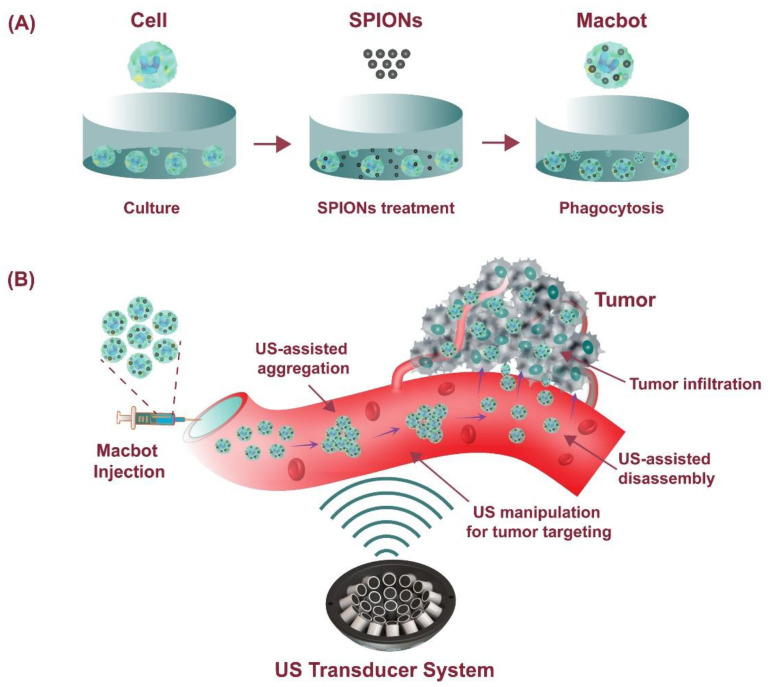
Schematic diagram showing the concept of using acoustically driven cell-based microrobots (Macbots) for targeted tumor therapy: (**A**) Fabrication process of the Macbots; (**B**) Working principle of the Macbots using ultrasonic actuator system.

In this study, we propose a cell-based microrobot system, which is wirelessly manipulated by external acoustic actuation. The system consists of a cell-based microrobot, an acoustic actuator system, and control methods to perform a four-degrees-of-freedom manipulation of the microrobot in water. The proposed acoustic actuator contains 30 ultrasonic transducers operating at 1 MHz and generates a twin-trap at the trap point. The distance between two cylindrical beams in the twin-trap is approximately 1.2 mm. Thus, the proposed system cannot trap and manipulate a single macrophage cell at the size of 21 μm. To trap the Macbots, superparamagnetic iron oxide nanoparticles (SPIONs) are used to assist the aggregation of macrophages. Figure S1 shows the result of trap and manipulation with only macrophages, only SPIONs, and Macbots. At the twin-trap point, the Macbot is aggregated and trapped as a cluster with approximate dimensions of 400 μm (width) × 800 μm (height) × 60 μm (depth). Without the SPIONs, the macrophages are separated and unstable in the trap point, thereby making it impossible to trap. We successfully demonstrated remote manipulation of a microrobot under closed-loop control to perform complex motion along a preprogrammed trajectory under the acoustic field in an in vitro environment. As a proof-of-concept evaluation for the targeted tumor therapy, two in vitro experiments were conducted in which a cell-based microrobot carrying SPIONs as a drug container was manipulated. In the first experiment, the acoustic actuation system controlled the cell-based microrobots following the spherical trajectory to find the optimal region of interest, retaining a position error of less than ±200 μm. The second experiment shows that the cell-based microrobot was manipulated along a complicated helical trajectory, and the position error of this experiment was 99.5 ± 82.7 μm on the Z-axis. Finally, the ability of microrobots to target and deeply penetrate a 3D tumor spheroid that mimics a solid tumor in an in vivo environment was verified. Figure 1 shows the schematic diagram of the concept of using acoustically driven cell-based microrobots for targeted tumor therapy.

## 2. Materials and Methods

### 2.1. Preparation of SPIONs

We prepared SPIONs using a co-precipitation technique based on a previously established protocol with some modifications [55]. The chemical reaction is shown in Equation (1):

2FeCl_3_ + FeCl_2_ + 8NH_3_ + 4H_2_O → Fe_3_O_4_ + 8NH_4_Cl (1)

For the reaction, FeCl_3_ and FeCl_2_ (provided by Sigma Aldrich, St. Louis, MO, USA) were dissolved in DI water in a three-necked flask prior and then heated to 80 °C with continuous stirring using a mechanical stirrer. After that, 20 mL of ammonia solution was slowly dropped into the mixture via a glass syringe. The reaction occurred in a nitrogen gas environment. The formed SPIONs were harvested using a permanent magnet and rinsed several times with DI water. Then, the clean SPIONs were dispersed in DI water, and 10 mL of citric acid (0.5 mg/mL) was added. The temperature of the solution was increased to 90 °C with continuous stirring for 60 min. After that, the harvested SPIONs coated with citric acid were harvested, rinsed with DI water, and stored at 4 °C for further experiments.

The surface morphology of the SPIONs was characterized using transmission electron microscopy (TEM, TECNAI F20 ST, FEI Company, Hillsboro, OR, USA). The average size of the SPIONs was calculated using a TEM image and processed with ImageJ software (ImageJ ver 1.53s, NIH, Bethesda, MD, USA).

### 2.2. Cell Culture

A raw 264.7 macrophage cell line was obtained from the Korea Cell Line Bank (Seoul, Korea). A549 cancer cells were purchased from the American Type Culture Collection (Manassas, VA, USA). These cells were grown in RPMI 1640 media supplemented with 10% fetal bovine saline (FBS) and 1% antibiotic solution in a humidified incubator at 37 °C with 5% CO_2_ [56].

### 2.3. Preparation of Macbots

Macrophages were plated in a 100 mm cell culture dish overnight with 5 × 10^7^ cells/dish. After that, the spent media were discarded and replaced with fresh media containing 0.5 mg/mL of SPIONs. The cells and SPIONs were co-incubated for 8 h to allow the phagocytosis of the SPIONs by the macrophages. Next, the media were discarded and replaced with phosphate-buffered saline (PBS). The washing process was conducted several times to completely remove the unphagocytosed SPIONs. The macrophages containing SPIONs, which were termed “microrobots” or Macbots, were harvested using a cell scraper [57].

The successful phagocytosis of SPIONs by the macrophages was verified using a TEM. After the Macbots were harvested as mentioned above, they were fixed in 2% glutaraldehyde overnight. After several washes with PBS, the Macbots were treated with 1% osmium tetroxide solution at 4 °C for 2 h. Then, the microrobots underwent three washes with PBS. Afterward, dehydration of the microrobots was performed with a series of ethanol solutions (50–100%) and a mixture of acetone/ethanol (1/1 *v*/*v*) for 20 min. The dehydrated Macbots were then immersed in a solution containing acetone/epoxide resin (1/2, *v*/*v*) overnight. Next, the Macbots were soaked in pure resin, allowing the polymerization process of the specimen. After that, the specimens were cut into 80–100 nm ultrathin slices using a diamond knife of ultra-microtome equipment. Finally, the slice was placed onto a carbon-coated grid before performing TEM [58].

### 2.4. Acoustically Driven Macbots

The movement of a particle in an acoustic field can be described by the following Equation [59,60,61]:

(2) $$m\ddot{x}=F_{g}+F_{rad}+F_{dag}+F_{bouyancy},$$

where *x* indicates the directional movement, *m* denotes the mass of the spherical particle, *F_g_* is a constant and is the gravity force acting on the spherical particle, *F_rad_* is the acoustic radiation force acting on the spherical particle in the acoustic field, *F_dag_* is the fluid drag force, and *F_bouyancy_* is the buoyant force. When fluid contains a particle suspension, *F_g_* and *F_bouyancy_* have constant values and cancel each other. Thus, the force effects on the movement of a particle are *F_rad_* and *F_dag_*.

In acoustic fields, the acoustic radiation force (*F_rad_*) exerted on the Rayleigh particle (radius of particle << wavelength of the acoustic wave) can be calculated from the gradient of the Gor’kov potential [62] following Equation (3):

(3) $$F_{rad}=-\nabla U$$

(4) $$U=2K_{1}\left({\left|p\right|}^{2}\right)-2K_{2}\left({\left|p_{x}\right|}^{2}+{\left|p_{y}\right|}^{2}+{\left|p_{z}\right|}^{2}\right)$$

(5) $$K_{1}=\frac{1}{4}V\left(\frac{1}{c_{0}^{2}\rho_{0}}-\frac{1}{c_{1}^{2}\rho_{1}}\right)$$

(6) $$K_{2}=\frac{3}{4}V\left(\frac{\rho_{0}-\rho_{1}}{\omega^{2}\rho_{0}\left(\rho_{0}+2\rho_{1}\right)}\right),$$

where *U* is defined for the Gor’kov potential; *x*, *y*, and *z* are the axes on the Cartesian coordinate; *P* is the complex acoustic pressure in the medium, and its spatial derivatives are written in Equation (4); *K*_1_ and *K*_2_ are the calculated coefficients, which are explained in Equations (5) and (6), respectively; *C* is the speed of sound; *ρ* is the density with the subscripts 0 and 1 representing the fluid and the particle material, respectively; and *V* is the volume of the spherical particles.

In this study, we offer a new technique for localized anti-tumor treatment. First, the macrophage cells and SPIONs were co-incubated to allow the phagocytosis of the SPIONs by the macrophages. Then, the macrophages containing SPIONs, termed “microrobots” or Macbots, were harvested. The Macbots were trapped and manipulated to deliver them to the edge of the tumor by the proposed ultrasound manipulation system. Thus, the SPIONs and macrophages were moved together under acoustic power. Finally, the macrophages can target and penetrate tumors by chemotaxis.

**Figure 2:**
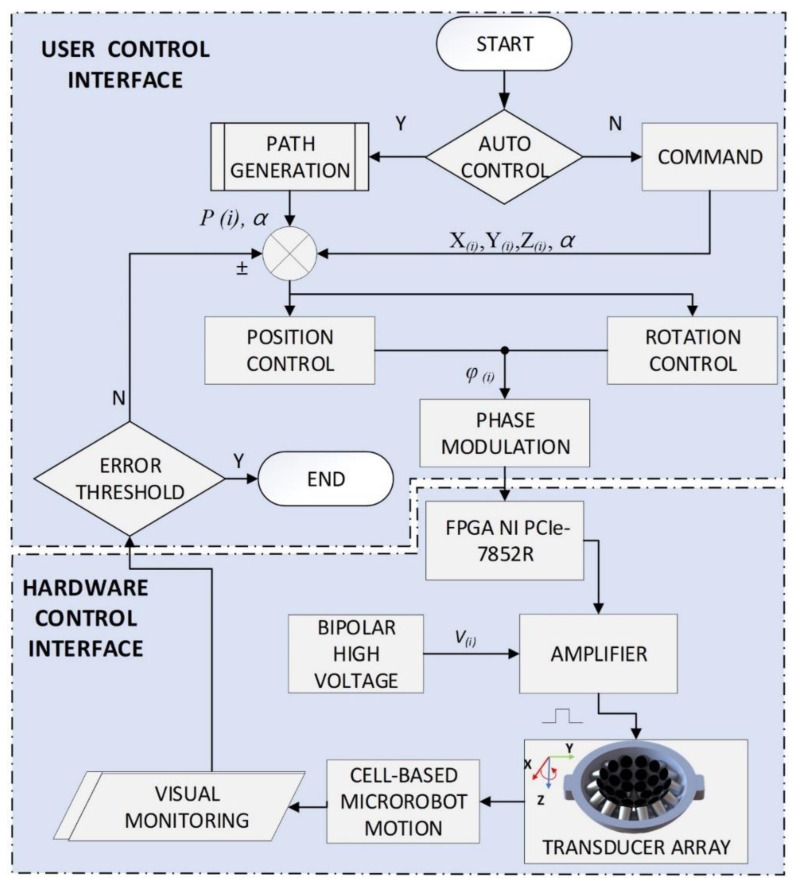
Close-loop control block diagram for acoustically driven Macbots experiment.

To manipulate the Macbots using an external acoustic field, we developed an acoustic actuator system consisting of three main subsystems: (1) a single-side ultrasound transducer (UT) array with 30 identical planar transducers of 16 mm diameter with individual focus from 18 mm to 32 mm (JAPAN PROBE, Yokohama, Japan); (2) a customized amplifier; and (3) a user control interface developed on LabVIEW 2017. The 30 transducers were operated at the same frequency of 1 MHz and the same operational bipolar voltage of 60 V from the P3030 power supply unit (Advantek, Hayward, CA, USA) but with independent phase control. The acoustic twin-trap is generated at the focus point by using the phase modulation method, which contains the twin-trap phase (with a π-radian difference on each side of the UT array) and the focus point position information. Thus, the function to control the position of the microrobot can be expressed by the following equations [7]:

(7) $$F_{rad}^{control}=\overset{i=30}{\underset{i=1}{f}}(\varphi_{i})$$

(8) $$\varphi_{i}=\left[\begin{array}{l} 0+2\pi (1-f_{i}(N))\;with\;i=1:1:15\\ \pi +2\pi (1-f_{i}(N))\;with\;i=16:1:30 \end{array}\right.,$$

where *φ_i_* is the phase delay in radian on the *i*th transducer; N is the number of acoustic wave cycles; and f*_i_*(N) is the decimal part of the last cycle. The customized amplifier was developed on the LabVIEW FPGA platform with FPGA PCIe-7852R hardware. The FPGA module generated the control signal at 3.3 V containing the phase value with a resolution of 2°. Then, the amplifier magnified the signal to 60 Vpp with retention of the phase information from the control signal in continuous mode. From the control interface, the wireless joystick was used to control the position of the microrobot with real-time videos from two charge-coupled device (CCD) cameras with 2.3 MP color blackfly PoE GigE C-mount (Teledyne FLIR, Wilsonville, OR, USA). Additionally, the close-loop control was implemented using the proportional control method (P-control) to improve the performance of the position control. Figure 2 shows a close-loop control block diagram for the acoustically driven Macbots experiment. The acoustic twin-trap generated at the initial focus point of (0, 0, 0) refers to the UT array coordinate to trap the Macbots. To manipulate the Macbot, the input data as 3D positions X_(*i*)_, Y_(*i*)_, Z_(*i*)_ and the angle α are required to calculate the phase delay for each transducer according to Equations (7) and (8). Then, by shifting the trap point, the position and angle of the Macbots move to the new trap point.

We also implemented the method to control the angle of Macbots in the O-XY plane. The proposed transducer array contains 30 transducers that can be set in 8 angular steps for the twin-trap phase. The Macbot is thus free to rotate in the O-XY plane by 360°/8 = 45° for each step [36]. The current position of the Macbots in the O-XY plane was captured by a CCD camera mounted on the top, whereas that of the Macbots in the O-XZ plane was captured by a CCD camera mounted on the side. LabVIEW Vision module with an object tracking function was used to calculate the current position of the Macbots. The current position of the Macbots was calculated by comparing the current frame captured by the camera with the background frame. On the basis of the current position, which was compared with the desired position, the control program regenerated the trapping point to minimize the position error to less than ±200 μm.

**Figure 3:**
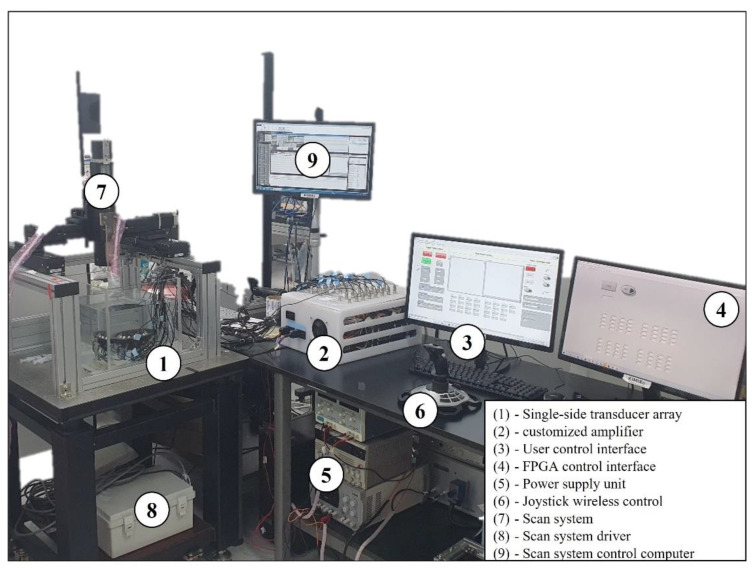
Experimental setup for the acoustically driven Macbots in water.

Figure 3 shows the experimental setup for the acoustically driven cell-based microrobots in the water tank with the size of 30 × 30 × 30 cm (approximation of the abdominal cavity). The experimental setup consists of nine subsystems: (1) a single-side transducer array, which was immersed in the water tank with a size of 30 × 30 × 30 cm (approximation of the abdominal cavity); (2) a customized amplifier; (3) a user control interface, which was developed on LabVIEW 2017; (4) an FPGA control interface, which was developed on LabVIEW FPGA 2017; (5) a power supply unit (Advantek, Hayward, CA, USA), which supplies regulated DC at 5 V, 3.3 V, and bipolar voltage (Exso, Busan, Korea) from ±5 V to ±30 V; (6) Joystick wireless control; (7) a three-dimension scan system; (8) a scan system driver; and (9) a scan system control computer.

### 2.5. Targeting the Macbots to 3D Tumor Spheroids

We prepared a tumor spheroid in vitro to mimic an in vivo solid tumor environment. To generate a tumor spheroid, we utilized a 96-well ultralow attachment cell culture plate (Corning). In brief, A549 tumor cells were harvested, counted, and re-suspended in the complete culture media. Then, 1 × 10^4^ cells in 100 μL media were placed in each well. After that, the plate was spun at 1200 rpm for 3 min. Then, the cells were maintained in an incubator for 7 days to allow the formation of tumor spheroids. After that, the spent media were changed, and the spheroids were treated with different samples including PBS, macrophage cells only, and the Macbots (1 × 10^4^ cells/microrobots per spheroid). For visualization under a confocal microscope system, the cells/Macbots were stained with CellTrace^TM^ Far Red (ThermoFisher, Waltham, MA, USA) before adding them to the spheroids. Penetration of the cells/Macbots into the spheroids was allowed for 12 h in an incubator at 37 °C. After 3 washes with PBS, the spheroids were fixed with 1% paraformaldehyde for 10 min, followed by a counterstain with 4,6-diamidino-2-phenylindole dihydrochloride (DAPI, 1 μg/mL). Finally, the spheroids were rinsed thrice with PBS, placed in a confocal dish, and observed under a confocal microscope system.

## 3. Results and Discussion

### 3.1. Preparation of SPIONs

**Figure 4:**
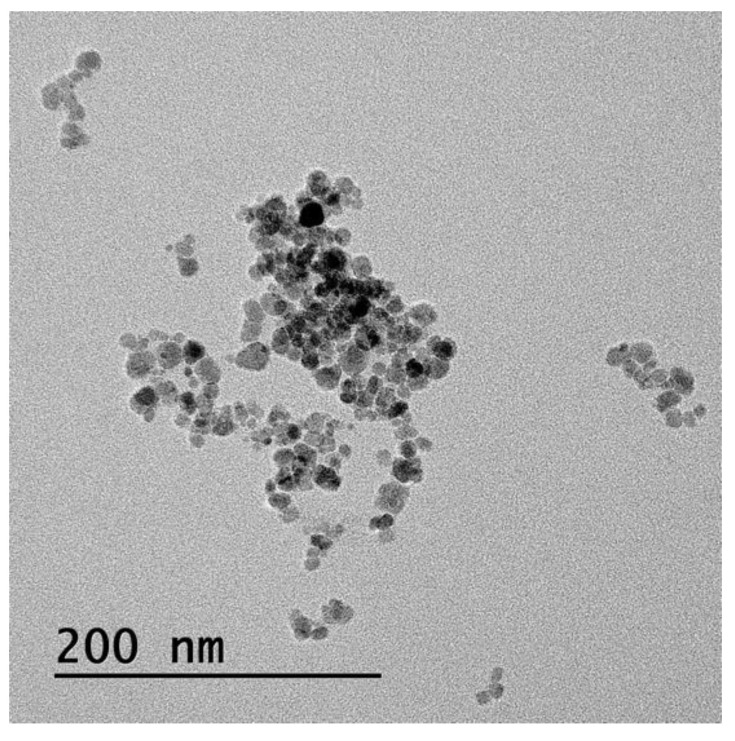
TEM image of SPIONs.

Figure 4 shows the TEM image of SPIONs prepared in this work using the chemical co-precipitation method. The SPIONs displayed spherical shapes with an average diameter of approximately 10 nm. Their magnetization value was 50.22 A × m^2^/kg (50.22 emu/g), thus illustrating that the prepared SPIONs possessed superparamagnetic properties.

### 3.2. Preparation of Macbots

**Figure 5:**
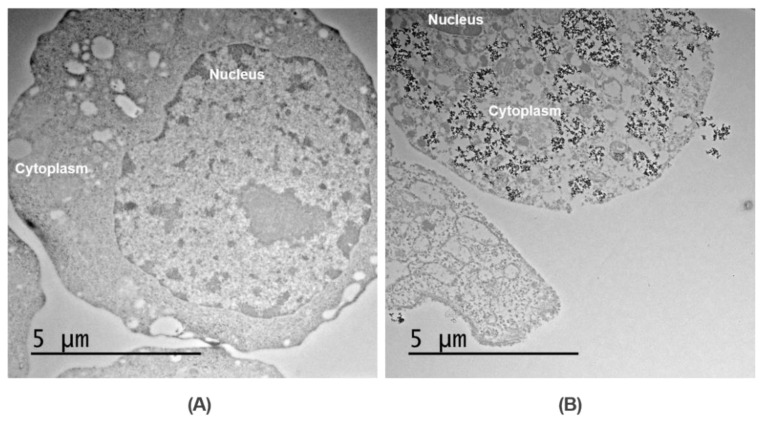
Bio-TEM image of a macrophage cell (**A**) and a Macbot (**B**).

The microrobots were prepared by the phagocytosis of the prepared citric-acid-coated SPIONs by macrophages. After 8 h of co-incubation of the macrophages with 0.5 mg/mL SPIONs, almost all cells engulfed the SPIONs. To verify the internalization of the prepared SPIONs to the macrophage cells, the cells were pretreated with chemicals. Then, the cells embedded in resin polymer were cut into ultrathin sections, which could be viewed using the TEM imaging system. Figure 5 shows the TEM images of two samples containing macrophage cells treated with PBS (Figure 5A) and SPIONs (the Macbot) (Figure 5B). A large amount of SPIONs (black dots) was engulfed by the macrophages and stayed in the cells’ cytoplasm without entering the nuclei, thus confirming the successful preparation of the Macbots.

### 3.3. Manipulation of Macbots Using the Acoustic Actuator System

**Figure 6:**
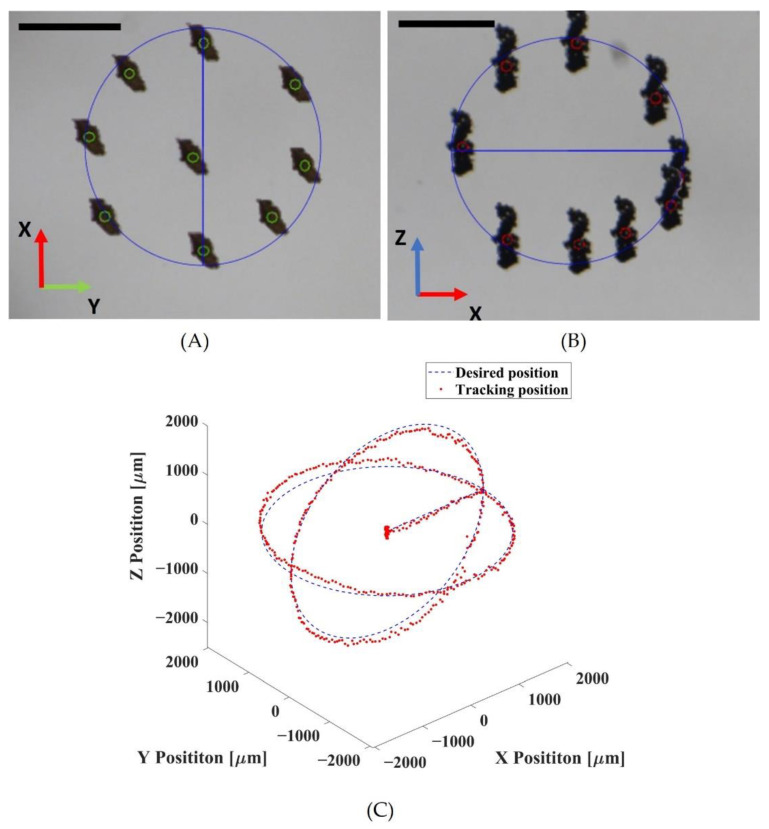
Demonstration of automated manipulation of Macbots following spherical trajectory: (**A**) time-lapse image sequence in O-XY view; (**B**) time-lapse image sequence in O-XZ view; (**C**) position tracking denoted by red dots corresponding to the desired position denoted by the blue dotted line (scale bar: 1 mm).

An experiment was conducted to validate the feasibility of acoustically driven Macbots in vitro. In the currently developed software, the system could manually generate a trajectory on the basis of the target position. Then, the Macbots were automatically manipulated following desired trajectories (e.g., spiral and helical ones), which were entered into the control system, thus allowing an operator not to follow a trajectory with the joystick. The Macbots were manipulated on two trajectories: spherical and helical. We performed the manipulation following the spherical trajectory in the first experiment to find the best performance workspace. The system could control the microrobots up to ±5 mm, ±5 mm, and ±4 mm in the X, Y, and Z directions, respectively. A spherical workspace with a diameter of 4 mm was selected to perform 3D automated manipulation with a position error of less than 300 μm. First, the Macbots were placed in the twin-trap point, and then the system started tracking and calculating their positions. After determining a stable position, the system manipulated the Macbot cluster to follow a spherical trajectory. The root mean square error (RMSE) and standard deviation (SD) in this trajectory were 118.7 ± 118.2 μm, 63.3 ± 60.8 μm, and 175.5 ± 82.7 μm on the X-, Y-, and Z-axes, respectively. Figure 6 shows the automated manipulation of the Macbots following spherical trajectory with a time-lapse image sequence and real-time tracking of the position of the microrobot.

**Figure 7:**
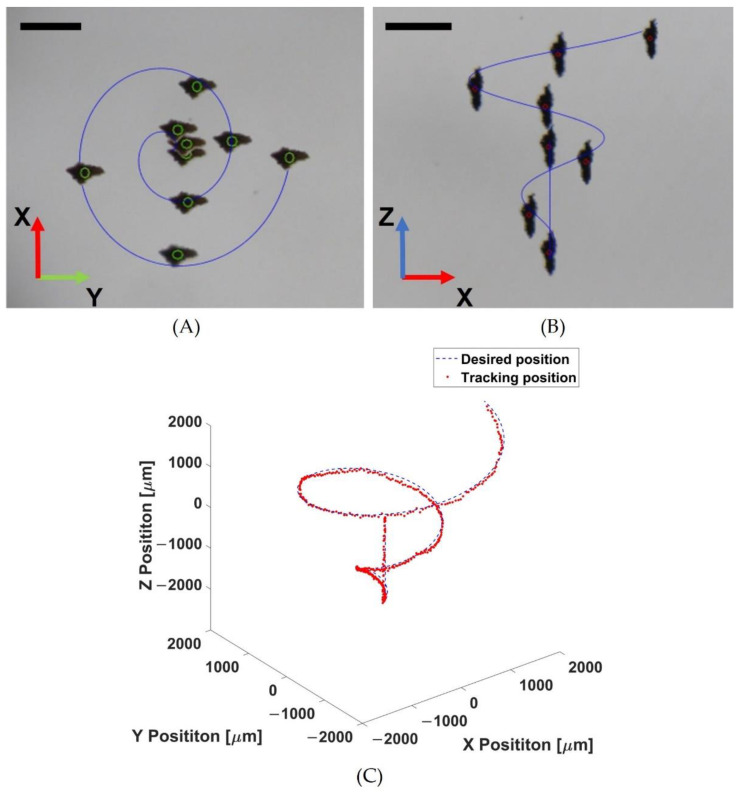
Demonstration of automated manipulation of Macbots following helical trajectory: (**A**) time-lapse image sequence in O-XY view; (**B**) time-lapse image sequence in O-XZ view; (**C**) position tracking denoted by red colored dots corresponding to the desired position denoted by the blue colored line (scale bar: 1 mm).

In the second experiment, the helical motion of the Macbots was performed, which was more complex than the spherical one. The Macbots were first trapped at the focus point and manipulated to the position of (0, 0, −2). Then, they were automatically controlled by following a helical trajectory with a maximum radius of 2 mm. The RMSE and SD in this trajectory were 63.5 ± 60.8 μm, 65.5 ± 63.2 μm, and 99.5 ± 82.7 μm on the X-, Y-, and Z-axes, respectively. Figure 7 shows the time-lapse image sequence of helical motion and tracking position with automated closed-loop position control and open-loop orientation control.

**Figure 8:**
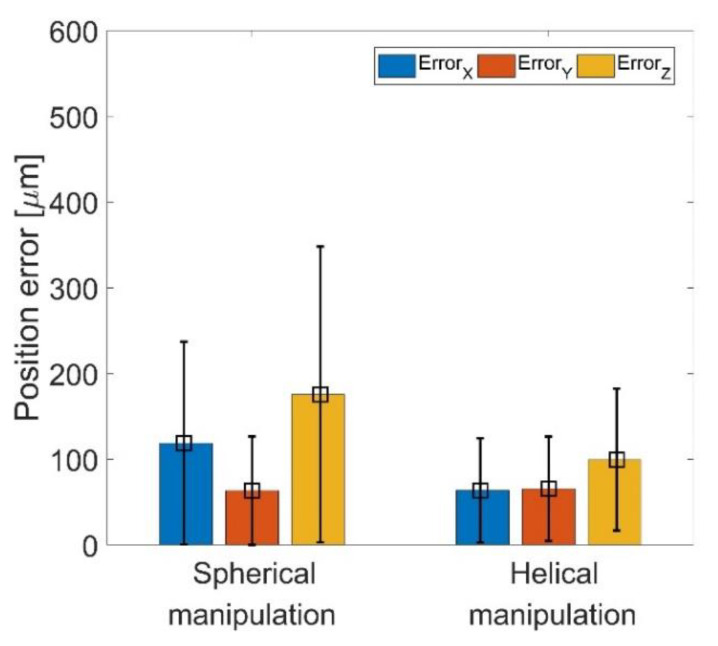
RMSE and SD of position error on the X-, Y-, and Z-axes in trajectory 1: spherical automated manipulation and trajectory 2: helical automated manipulation.

Figure 8 shows the RMSE and SD of position error in the X, Y, and Z directions for both spherical and helical trajectories. The position error on the Z-axis was most significant at 175.5 ± 82.7 μm in the spherical trajectory. The result is in agreement with the characteristic of the acoustic twin-trap in that the acoustic radiation force on the Z-axis was the weakest [34]. The position errors in the spherical trajectory were larger than those in the helical trajectory, which means that the acoustic radiation force decreased when the twin-trap was electrically regenerated at a distant physical focal point.

### 3.4. Evaluation of Tumor Targeting of Macbots

We evaluated the tumor targeting of the prepared Macbots by assessing their ability to penetrate a 3D tumor spheroid to mimic an in vivo solid tumor. As a part of the mononuclear phagocyte system, macrophages can target and penetrate tumors through chemotaxis. To evaluate the chemotaxis of cells, many well-established setups, such as microfluidic channel [63], Boyden chamber [6], and scratch assay [52], have been utilized. However, these experimental setups are usually composed of a monolayer of cancer cells, which provides insufficient drug and oxygen diffusion gradients, resulting in poor therapeutic response. Although 3D tumor spheroid models do not perfectly resemble the real in vivo environment, they represent the condition better than the above-mentioned setups [5].

**Figure 9:**
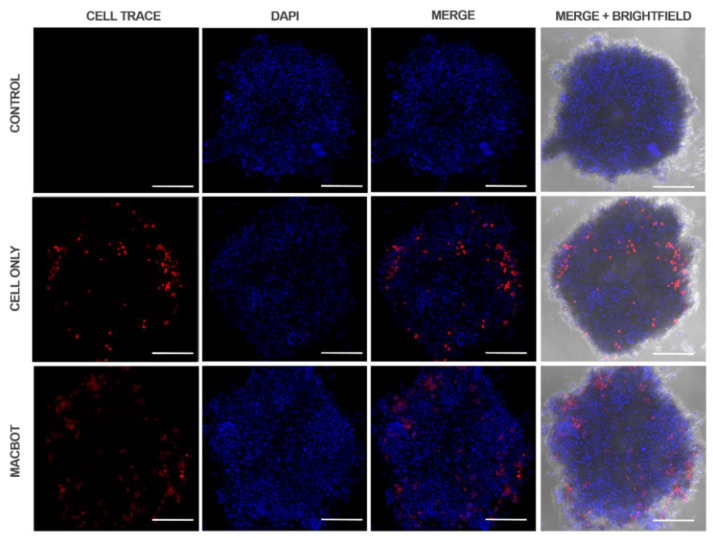
Penetration of the macrophage cells/Macbots into A549 tumor spheroids. The macrophages and microrobots were stained with CellTrace^TM^ Far Red (red), and the spheroids were counterstained with DAPI (blue). Scale bars 200 μm.

Figure 9 shows the penetration of the cells and the Macbots into A549 tumor spheroids. The macrophage cells could deeply infiltrate the tumor spheroid, as indicated by the red signal of the macrophage stain. Similar results were observed after treating the spheroids with the Macbots. Thus, even after being engulfed by SPIONs, the macrophages retained the ability to penetrate the tumor spheroids. The penetration efficiency of different samples was quantified and compared by determining the ratio of the red signal area (CELL TRACE) showing the signals of the cells/Macbots and the entire red plus blue signal area (CELL TRACE + DAPI). For that, the images were analyzed, the signal channels were extracted using ImageJ (NIH), and the area of each channel and the total area were calculated (Figure S2). The “penetration index” was determined using the following Equation (9):

(9) $$\mathrm{Penetration}\;\mathrm{index}\;\left(\%\right)=\frac{\mathrm{Red}\;\mathrm{area}}{\mathrm{Red}\;\mathrm{area}+\mathrm{Blue}\;\mathrm{area}}\;\times \;100$$

The penetration indices of the cells and Macbots were calculated to be 11.37% and 17.90%, respectively.

Figures S3–S5 show the penetration of the control, macrophage cells, and the Macbots at different cutting planes of the spheroids, respectively. The red signals of the macrophages/Macbots were clearly observed in different depths of the spheroid, thus indicating that the cells/Macbots did not merely attach to the surfaces of the spheroid but could efficiently infiltrate the spheroids.

## 4. Conclusions and Future Work

We developed a novel acoustic manipulation system and macrophage-based microrobots for a feasibility study in targeted tumor therapy. We demonstrated that the Macbots with loaded SPIONs could be trapped and manipulated using the acoustic actuator system as the cluster. The microrobot cluster could be manipulated to any point in an ROI of 4 × 4 × 4 mm. The RMSE and SD were 63.5 ± 60.8 μm, 65.5 ± 63.2 μm, and 99.5 ± 82.7 μm for the helical trajectory, and 118.7 ± 118.2 μm, 63.3 ± 60.8 μm, and 175.5 ± 82.7 μm for the spherical trajectory on the X-, Y-, and Z-axes, respectively. The most significant position error was on the Z-axis, and the smallest was on the X-axis. Furthermore, the microrobot cluster could rotate in the O-XY plane with an angle step of 45° without limitation of the total angle. Finally, it was demonstrated that the Macbots manifested their ability to penetrate a 3D tumor spheroid to mimic an in vivo solid tumor.

Although the system developed in this study shows promising potential for application in targeted tumor therapy, the study has some limitations. The experiments in this study were conducted in water without considering the flow conditions and high viscosity in actual biological environments. Therefore, in future studies, we plan to verify the performance of the proposed system for application to clinical sites through animal experiments as well as by considering the actual environment. In addition, it is necessary to know the amount of SPIONs in each treatment group to compare the therapeutic effects; for this, we will verify the quantified amount of SPIONs in macrophages when conducting cell and animal experiments. Furthermore, in this study, the acoustic actuation manipulated the Macbots to the edge of the tumor. Then, macrophages can target and penetrate tumors driven by chemotaxis. In the current report, we did not evaluate the radiation force to the penetration of the Macbots. In a future study, we will improve the acoustic actuation, then investigate how the acoustic radiation force assists the penetration of the Macbots to tumors.

## Figures and Tables

**Table 1:** Summary of external powered platforms for wireless particle manipulation.

Actuation Power Platform	Single Object Size Range	Input Power	Control Force ^1^	Additional System Requirements
Acoustic field [7,34,35,36]	0.1–1000 (μm)	10^−2^–10 (W/cm^2^)	Axial acoustic force (μN)	Low acoustic impedance media, matching layer
Optical field [19,20,21,35]	0.1–100 (μm)	10^6^–10^7^ (W/cm^2^)	Trapping force and torque (pN)	High numerical aperture lens, high-powered laser system.
Magnetic field [22,23,24,35]	0.1–10 (μm)	1–10 (Tesla)	Magnetic gradient field force (nN to μN)	Magnetic particles, high-current power source
Hydrodynamic field [28,29,30,35]	0.1–1100 (μm)	N/A	Hydrodynamic effects (pN to μN)	Flow control method, specific platform
Electric field [31,32,33]	0.001–1000 (μm)	10^4^–10^7^ (V/m)	Dielectrophoresis force (pN to μN)	Low-conductivity media, AC electric signal

^1^ The range of control force is given for specialized conditions.

## Supplementary Materials

The following supporting information can be downloaded at: https://www.mdpi.com/article/10.3390/pharmaceutics14102143/s1, Figure S1. Demonstration of trap and manipulation: (A) macrophage; (B) SPIONs; (C) Macbots. Figure S2. CELL TRACE and DAPI channels from the merged images were extracted for quantitative analysis of penetration. Figure S3. Penetration into A549 tumor spheroids imaged at different depths of the spheroids of the control sample. Scale bars 200 μm. Figure S4. Penetration into A549 tumor spheroids imaged at different depths of the spheroids of the cell-only sample. Scale bars 200 μm. Figure S5. Penetration into A549 tumor spheroids imaged at different depths of the spheroids of the Macbot sample. Scale bars 200 μm.
