# Supplementary material for: Acoustically Driven Cell-Based Microrobots for Targeted Tumor Therapy
Source: Pharmaceutics. 2022 Oct 9;14(10):2143. doi: 10.3390/pharmaceutics14102143 (PMC9609374; doi:10.3390/pharmaceutics14102143)
Supplement: Supplementary file 1 [file pharmaceutics-14-02143-s001.zip › pharmaceutics-1882562-supplementary.pdf]

pharmaceutics

Supplementary Materials

Acoustically Driven Cell-Based Microrobots for Targeted Tumor Therapy

Hiep Xuan Cao [1,2], Van Du Nguyen [1,2], Daewon Jung [2], Eunpyo Choi [1,2], Chang-Sei Kim [1,*], Jong-Oh Park [2,*], Byungjeon Kang [2,3,4,*+]

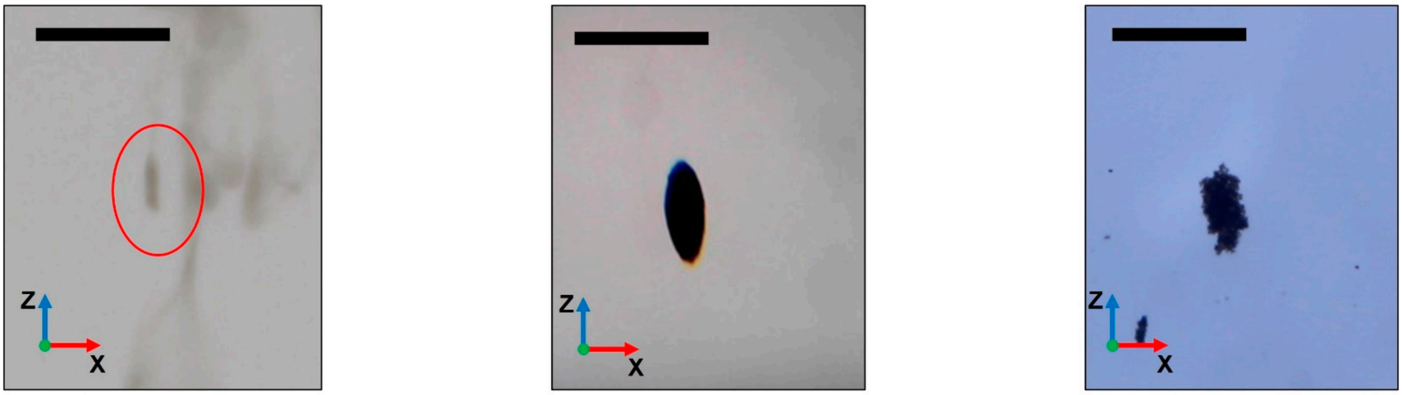

(A) (B) (C)

Figure S1. Demonstration of trap and manipulation: (A) macrophage; (B) SPIONs ; (C) Macbots.

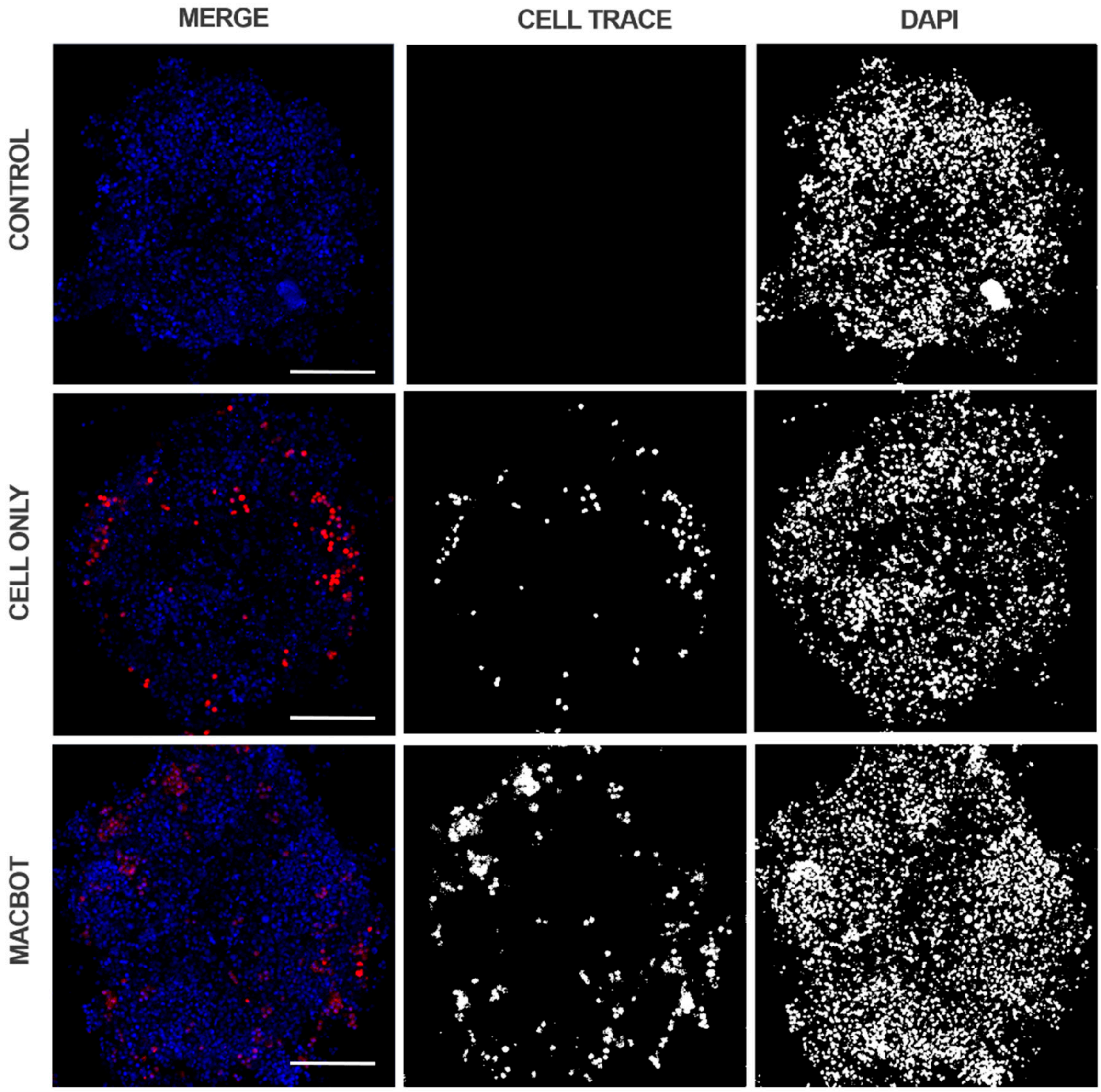

Figure S2. CELL TRACE and DAPI channels from the merged images were extracted for quantitative analysis of penetration.

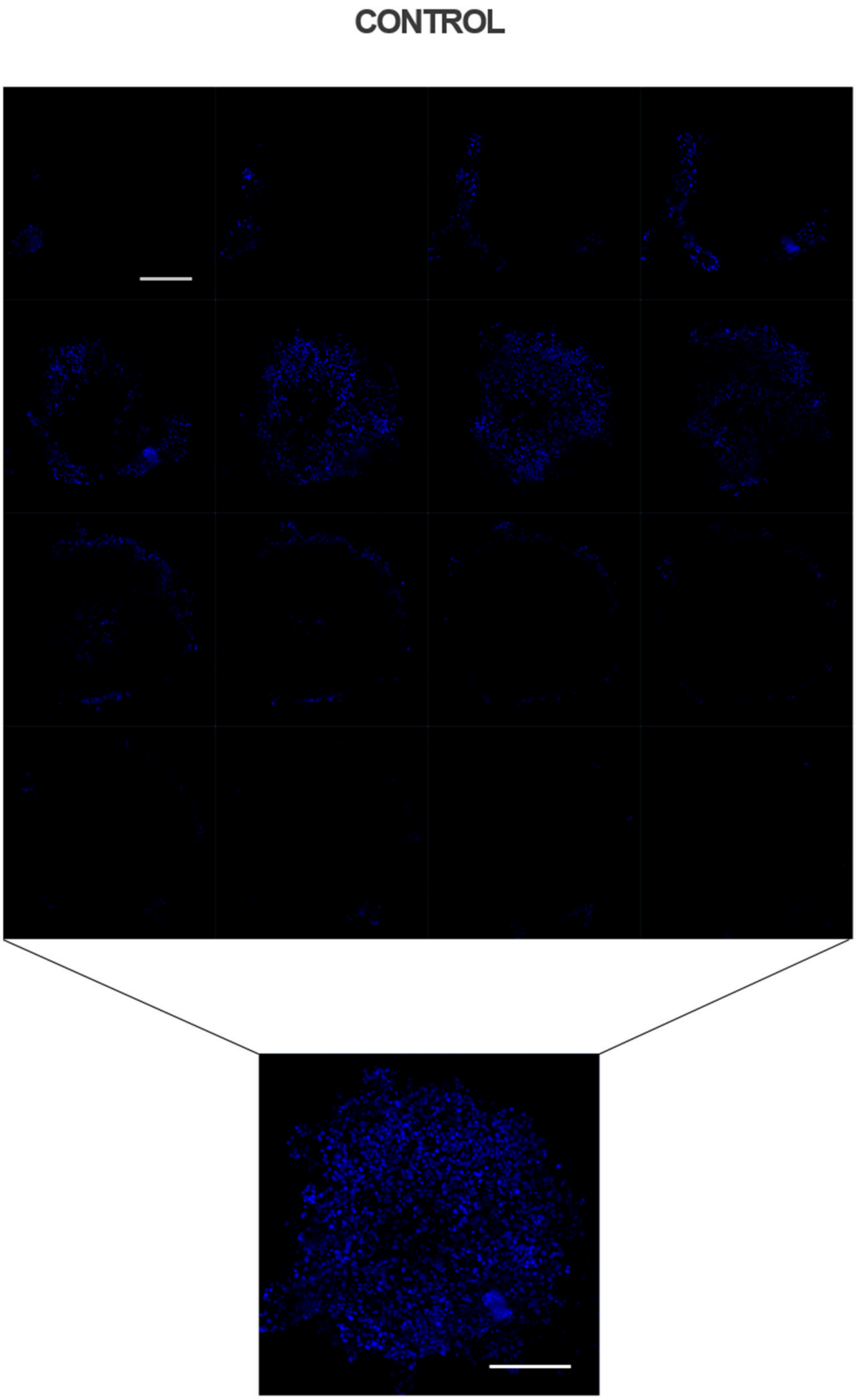

Figure S3. Penetration into A549 tumor spheroids imaged at different depths of the spheroids of Control sample. Scale bars 200 µm.

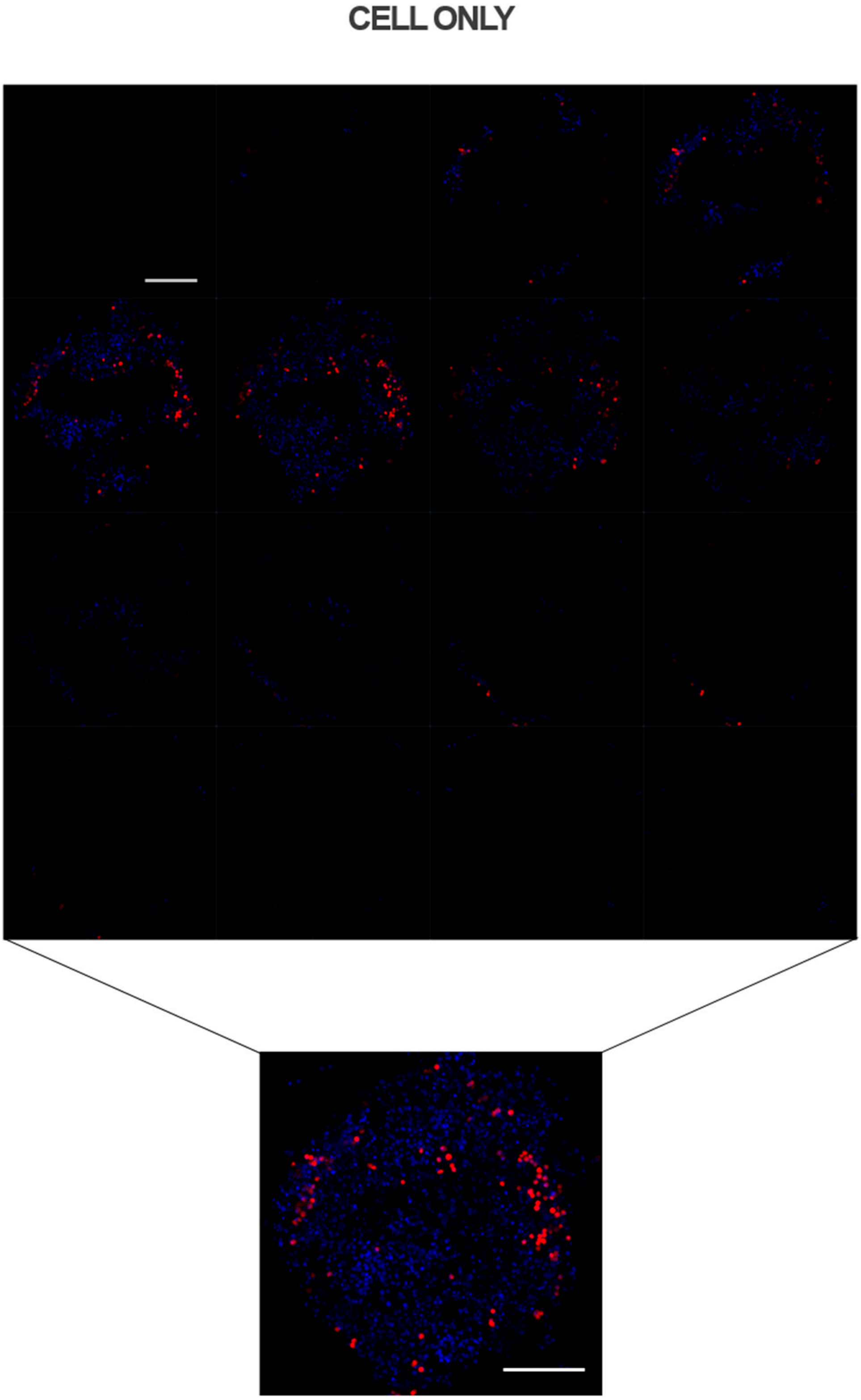

Figure S4. Penetration into A549 tumor spheroids imaged at different depths of the spheroids of Cell only sample. Scale bars 200 μm.

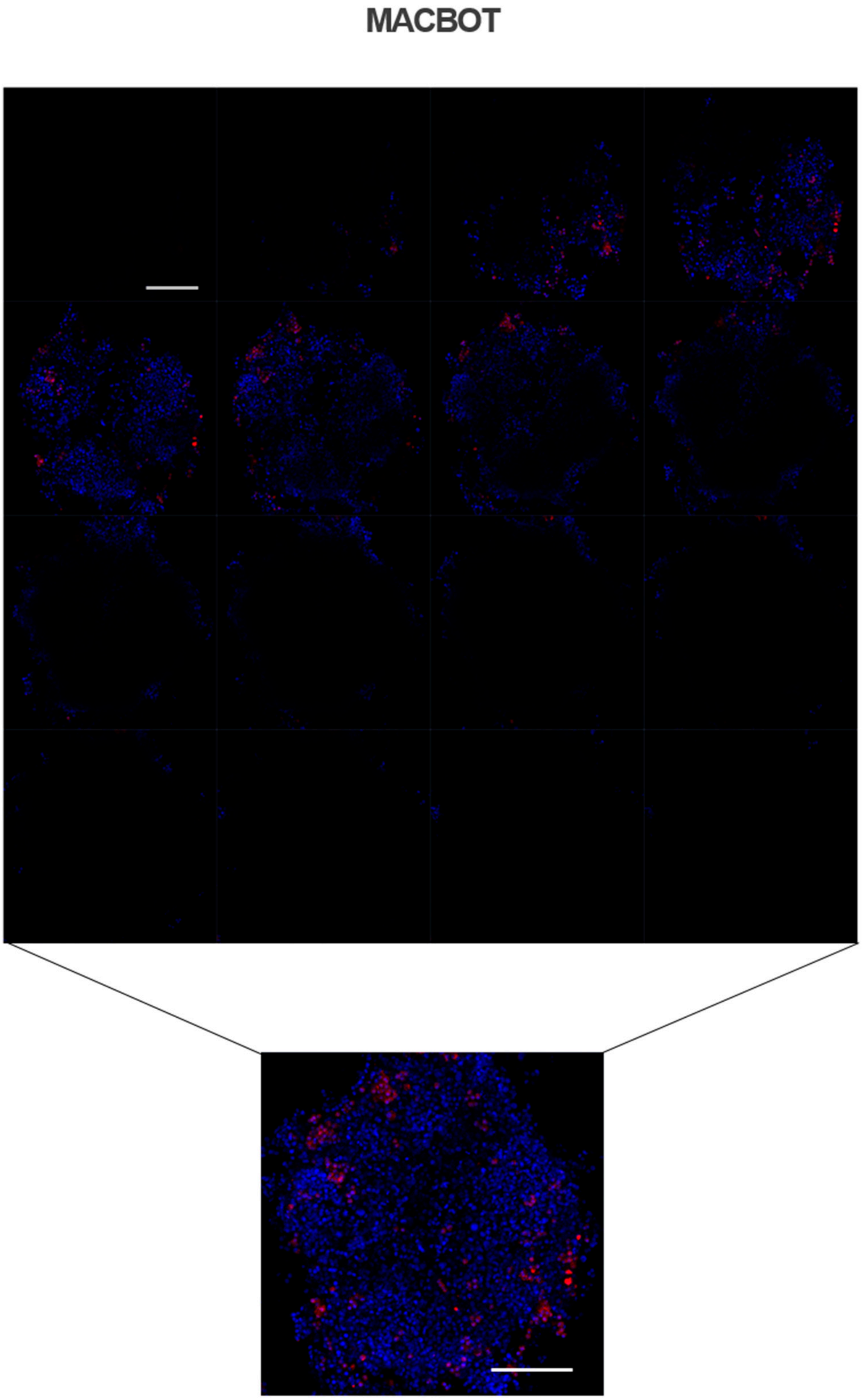

Figure S5. Penetration into A549 tumor spheroids imaged at different depths of the spheroids of Macbot sample. Scale bars 200 µm.
